# Detection of microplastics in human tissues and organs: A scoping review

**DOI:** 10.7189/jogh.14.04179

**Published:** 2024-08-23

**Authors:** Nur Sakinah Roslan, Yeong Yeh Lee, Yusof Shuaib Ibrahim, Sabiqah Tuan Anuar, Ku Mohd Kalkausar Ku Yusof, Lisa Ann Lai, Teresa Brentnall

**Affiliations:** 1 Faculty of Science and Marine Environment, Universiti Malaysia Terengganu, Kuala Nerus, Terengganu, Malaysia; 2 School of Medical Sciences, Universiti Sains Malaysia, Kota Bharu, Malaysia; 3 Microplastic Research Interest Group (MRIG), Faculty of Science and Marine Environment, Universiti Malaysia Terengganu, Kuala Nerus, Terengganu, Malaysia; 4 University of Washington, Seattle, Washington, USA

## Abstract

**Background:**

Research on microplastics has largely focused on the environment and marine organisms until recently. A growing body of evidence has detected microplastics in human organs and tissues, with their exact entry routes being unclear and their potential health effects remain unknown. This scoping review aimed to characterise microplastics in human tissues and organs, examine their entry routes and addressing gaps in research analytical techniques.

**Methods:**

Eligibility criteria included English language full text articles, in-vivo human studies only, and searching the databases using pre-defined terms. We based our analysis and reporting on the PRISMA guideline and examined the quality of evidence using the risk of bias assessment tool.

**Results:**

Of 3616 articles screened, 223 evaluated and 26 were eventually included in this review. Nine were high risk for bias, three were unclear risk and the rest low risk for bias. Microplastics were detected in 8/12 human organ systems including cardiovascular, digestive, endocrine, integumentary, lymphatic, respiratory, reproductive and urinary. Microplastics were also observed in other human biological samples such as breastmilk, meconium, semen, stool, sputum and urine. Microplastics can be characterised based on shape, colours, and polymer type. Potential entry routes into human included atmospheric inhalation and ingestion through food and water. The extraction techniques for analysis of microplastics in human tissues vary significantly, each offering distinct advantages and limitations.

**Conclusions:**

Microplastics are commonly detected in human tissues and organs, with distinct characteristics and entry routes, and variable analytical techniques exist.

The global production of plastics in 2020 alone is estimated at 367 million metric tons [1]. Mismanaged plastic wastes may lead to the formation of tiny plastics with the size of less than five mm, called as microplastics, into the environment via wind and water runoff [2]. These plastics can be broken down via weathering processes such as mechanical fragmentation, photo-degradation, thermal degradation, and biodegradation [3,4]. Microplastics are categorised into primary and secondary microplastics [5]. Primary microplastics, such as microbeads in cosmetics and microfibres from synthetic textiles, are intentionally manufactured at small size. Secondary microplastics, in contrast, are the result of the degradation and fragmentation of larger plastic items due to weathering processes. Microplastics are documented widely in aquatic and marine environments [6,7] and can be ingested by marine organisms, including fish [8], mussels [9] and shellfish [10], causing bioaccumulation and biomagnification. While the effects on environment and marine organisms have been extensively studied, similar studies in humans are lacking, with variable characteristics being reported in different studies. The variation in characteristics may be due to gaps and differences in research and analytical techniques [11,12]. Only recently that microplastics are being increasingly detected in various human organs, raising concerns about their health effects. Mechanism for health effects is unclear but microplastics may act as carriers for harmful chemicals and pathogen from the environment into human body. Therefore, this scoping review aimed to characterise microplastics in human tissues and organs, identify their entry routes, and identify gaps in analytical methodology. There may be discussion on potential health effects but these are by no means regarded as definitive due to limitations in current evidence.

## METHODS

### Study design

This study was formulated following the guidelines provided by the Preferred Reporting Items for Systematic Review and Meta-analysis extension for Scoping Reviews (PRISMA-ScR) (Table S1 in the **Online Supplementary Document****).**

### Inclusion and exclusion criteria

Inclusion criteria include English language full text articles focusing on in-vivo human studies published until 2024. Boolean operators such as ‘AND’ and ‘OR’ were employed effectively during the study selection alongside the keywords of ‘microplastics in human’, ‘human organ’, ‘tissues’ and ‘cancer' to prevent data oversaturation and enhance the precision of the retrieved information. Initial screens were conducted using the PubMed and Web of Science databases and detailed search strategy is shown in Appendix S1 in the **Online Supplementary Document**. Results of searches were exported into Mendeley and duplicates removed through Excel. Exclusion criteria included in vitro human studies or laboratory testing. Commentaries, opinion pieces, reviews, editorials and non-peer-reviewed reports were also excluded.

### Data extraction and collection

Three authors (NSR, YSI and LYY) worked on data extraction and initial draft. For each study, information such as type of organ, sample size, abundance, size, shape, colour and polymer composition of microplastics were extracted by the authors. Discrepancies among the authors were resolved through discussion and consensus. If disagreements persisted, a fourth author (STA or LAL) was consulted to make the final decision.

### Quality assessment

Risk of bias, methodology quality and reliability were determined using the Risk of Bias (RoB) assessment tool [13]. The tool was based on four domains - study design, sampling, analysis, and reporting (Table S2 in the **Online Supplementary Document**) [14–16]. The RoB tool also yields three ratings: high risk, low risk, or unclear risk. High risk refers to studies that met the domain criteria but obtained negative result. Low risk indicates studies that thoroughly addressed each domain, while studies that does not elaborate further on any of the domains are categorised as having an unclear risk.

### Patient and public involvement

No patient involved.

## RESULTS

**Figure 1:**
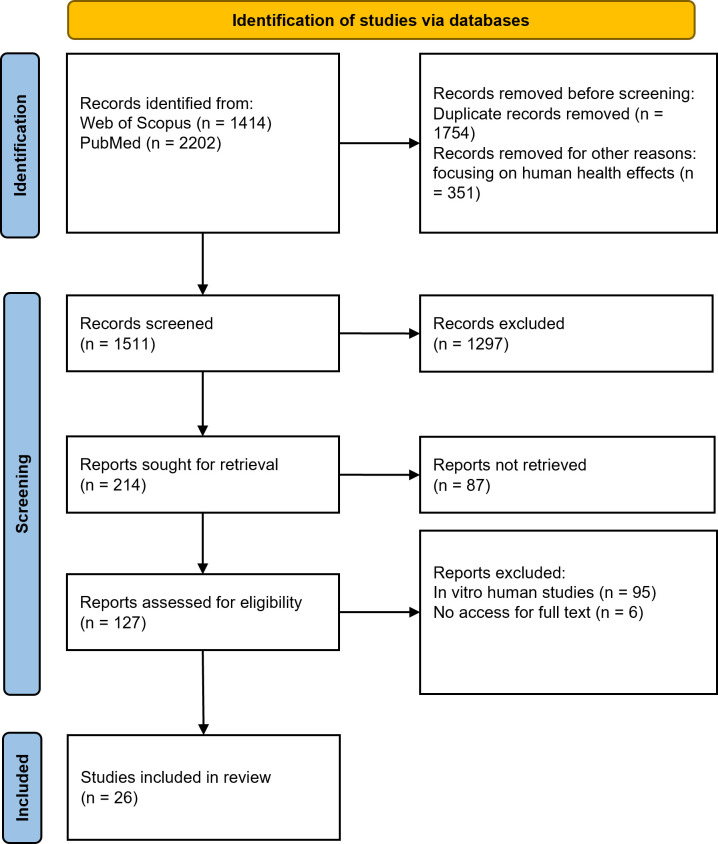
Literature screening flow.

**Figure 2:**
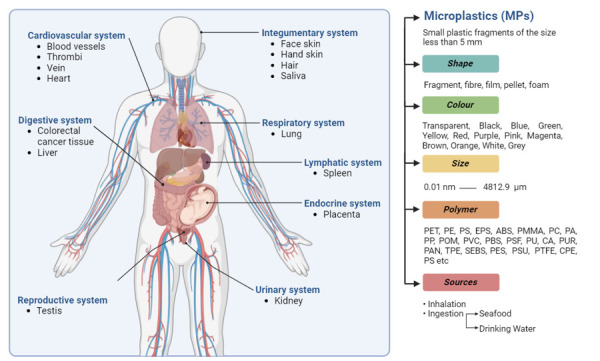
Summary of presence of microplastics in human body systems including their characteristics and possible pathway of microplastics into the body. Schematic representations were generated by BioRender.com. ABS – Acrylonitrile Butadiene Styrene, CA – Cellulose Acetate, CPE – Chlorinated Polyethylene, EPS – Expanded Polystyrene, mm – millimetre, PA – Polyamide, PAN – Polyacrylonitrile, PBS – Phosphate-buffered Saline, PES – Polyethersulfone, PE – Polyethylene, PET – Polyethylene Terephthalate, PC – Polycarbonate, PMMA – Polymethyl Methacrylate, POM – Polyoxymethylene, PP – Polypropylene, PS – Polystyrene, PSF/PSU – Polysulfone, PU/PUR – Polyurethane, PTFE – Polytetrafluoroethylene, PVC – Polyvinyl Chloride, TPE – Thermoplastic Elastomers, SEBS – Styrene-Ethylene-Butylene-Styrene, μm – micrometre.

A total of 3616 articles were initially identified. After screening using predetermined criteria, 223 articles were evaluated and 26 were finally included in the study (**Figure 1**). Our human body is composed of 12 organ systems, and eight of them have evidence of contamination by microplastics (**Figure 2**). These organ systems are cardiovascular system [17–20], digestive system [21,22], endocrine system [23–26], integumentary system [27], lymphatic system [22], respiratory system [28–30], reproductive system [31], and urinary system [22] (**Table 1**). In addition to organ systems, microplastics were also reported in other biological human samples such as breastmilk [26,32], meconium [24,26], infant faeces [26], semen [31,33], stool [34–39], sputum [40], and urine [41,42] (**Table 2**). Microplastics can be further categorised based on origin, morphology, colours, and polymer type (**Table 1**, **Table 2**). In addition, we found that atmospheric inhalation and ingestion through food and water were the likely primary routes of entry of microplastics into human body. Furthermore, the extraction methodologies for microplastics in human organs vary significantly, each offering distinct advantages and limitations. These diverse methodologies are comprehensively detailed in **Table 3**.

**Table 1:** Abundance of microplastics in human organ systems

System	Organ	Sample Size	Abundance of microplastics	Size of Microplastics	Shape of Microplastics	Colour of microplastics	Polymer of Microplastics	References
Cardiovascular system	Blood vessels	22	1.6 ug/mL	>700 nm	NA	NA	PET, PE, PS, EPS, ABS, PMMA	[17]
	Thrombi	26	87 particles	2.1–26.0 μm	Block shaped	Yellow, green, red	LDPE, Pigment, Chromium Oxide, Phthalocyanine	[18]
	Vein	5	20 particles or 14.99 ± 17.18 microplastic/g of tissue	16–1074 μm	Fragment, fibre	NA	Alkyd Resin, Poly(vinyl propionate), Nylon-ethylene-vinyl acetate, nylon-EVA, tie layer	[19]
	Heart	15	NA	20–500 μm	NA	NA	PET, PVC, PMMA	[20]
Digestive system	Colorectal cancer tissue	11	331 Microplastics per individual or 28.1–15.4 particles/g tissue	0.8–1.6 mm	Fibre	Transparent, black, red, green, blue, brown, purple, and yellow	PC, PA, PP	[21]
	Liver	11	0–13 particles per sample or 3.2 particles/g tissue	4–30 μm	Fragment, microbead	NA	PS, PVC, PET, PMMA, POM, PP	[22]
Endocrine system	Placenta	NA	12 particles	>5 μm	Fragment	Blue, purple, pink, orange, red	PP	[23]
	Placenta		NA	>50 μm	NA		PE, PP, PU	[24]
	Placenta	17	149 microplastics particles	20.34–307.24 μm	Fragment, fibre, film, subspherical particle	NA	PVC, PP, PBS, PET, PC, PS, PA, PE, PSF	[25]
	Placenta	18	NA	20–500 μm	NA	NA	PU, PA, PE, PET, PC	[26]
Integumentary system	Face skin	2000	4265 microplastics particles	100–500 μm	Spheres fragment, film, fibre	Blue, red, yellow, transparent, black	PE, PET, PS, PVC	[27]
	Hand skin	2000	4051 microplastics particles	100–500 μm	Sphere, fragment, film, fibre	Blue, red, yellow, transparent, black	PE, PET, PS, PVC	[27]
	Hair	2000	7462 microplastics particles	100–500 μm	Sphere, fragment, film, fibre	Blue, red, yellow, transparent, black	PE, PET, PS, PVC	[27]
	Saliva	2000	645 microplastics particles	100–500 μm	Sphere, fragment, film, fibre	Blue, red, yellow, transparent, black	PE, PET, PS, PVC	[27]
Lymphatic system	Spleen	3	4 particles per sample or 1.1 particles/g tissue	5–25 μm	Fragment, Microbead	NA	PS, PVC, PET, PMMA, POM, PP	[22]
Respiratory system	Lung tissues	20	31 particles	1.6–16.8 μm	Fragment, fibre	Transparent, white, blue, grey, yellow, brown, orange	PP, PE, Cotton, PVC, CA, PA, PS, PU	[28]
	Lung granule nodules	100	65 particles	>20 μm	Fibre	Purple, blue, transparent, yellow, red	Cotton, PA, Polyester, Denim, Phenoxy resin,	[29]
	Lung tissue	13	39 particles	12–2475 μm	Fibre, fragment, film	NA	PP, PET, Resin, PE, PTFE, PS, PAN, PES, PMMA, PUR, SEBS, TPE	[30]
Reproductive system	Testis	6	31 particles in 4 of 6 testis samples	20–100 μm	Fragment, fibre, film, subspherical	NA	PS, PVC, PE, PP	[31]
Urinary system	Kidney	3	0 particle per sample	10–20 μm	NA	NA	NA	[22]

ABS – Acrylonitrile Butadiene Styrene, CA – Cellulose Acetate, CPE – Chlorinated Polyethylene, EPS – Expanded Polystyrene, EVA – Ethylene-Vinyl Acetate, HDPE – High-Density Polyethylene, LDPE – Low Density Polyethylene, NA – not available, NC – Nitrocellulose, mm – millimetre, PA – Polyamide, PAN – Polyacrylonitrile, PBS – Phosphate-buffered Saline, PBT – Persistent Bioaccumulative Toxic, PES – Polyethersulfone, PET – Polyethylene Terephthalate, PEMA – Phenylethylmalonamide, PC – Polycarbonate, PLA – Polylactic acid, PMMA – Polymethyl methacrylate, POM – Polyoxymethylene, PP – Polypropylene, PS – Polystyrene, PSF/PSU – Polysulfone, PU/PUR – Polyurethane, PTFE – Polytetrafluoroethylene, PVC – Polyvinyl Chloride, PVOH – Polyvinyl Alcohol, TPE – Thermoplastic Elastomere, SEBS – Styrene-Ethylene-Butylene-Styrene, μm – micrometre, μg/mL – microgram per millilitre

**Table 2:** Abundance of microplastics in human biological samples

Type of sample	Sample Size	Abundance of microplastics	Size of microplastics	Shape of microplastics	Colour of microplastics	Polymer of microplastics	References
Breastmilk	7	20.2 particles/g	>20 μm	NA	NA	PA, PU, PE, PET, PP, PVC, POM, EVA, PTFE, CPE, Polybutadiene, PS, PMMA, PLA, Polysulfones	[26]
	34	58 particles in total	1–12 μm	Fragment, sphere	Orange, blue, black, red, grey, brown, green, transparent, magenta	PE, PVC, PP, CPE, PVOH, PEVA, PEMA, ABS, PES, PA, PC, PS, NC	[32]
Meconium	2	NA	>50 μm	NA	NA	PE, PP, PS	[24]
	12	54.1 particles/g	>20 μm	NA	NA	PA, PU, PE, PET, PP, PVC, POM, EVA, PTFE, CPE, PS, PMMA, PLA, Polysulfones	[26]
Infant faeces	12	26.6 particles/g	>20 μm	NA	NA	PA, PU, PE, PET, PP, PVC, POM, EVA, PTFE, CPE, Polybutadiene, PS, PMMA, PLA, Polysulfones	[26]
Semen	25 semen samples	24 microplastics in 11 of 25 semen samples (0.23 ± 0.45 particles/mL)	21.76–286.71 μm	Fibre, fragment, subspherical, film	NA	PVC, PE, PA, PP, PS, PET	[31]
	10 healthy young men	16 microplastics in 6 of 10 semen samples	2–5 μm	Fragment, sphere	Green, black, grey, orange, clear, yellow, blue, magenta	PP, PS, PET, PVS, PC, POM, Arcylic	[33]
Stool	8 healthy young men	9 particles in total	50–500 μm	Fragment, film	NA	PP, PET, PS, PE, POM, PC, PA, PVC, PU	[34]
	8 participants	129 particles (20.4–138.9 particles/g)	40.2–4812.9 μm	Fragment, fibre	NA	PS, PP, PE, PET, PVC	[35]
	50 of healthy adult	3070 particles (28 items/g)	4.4–333.2 μm	Sheet, fibre fragment, pellet	NA	PET, PA, PP, PE, PC, PVC, POM, PTFE, EVA, PS, PMMA, PBT, AS, PET, TPU	[36]
	52 of inflammatory bowel disease patients	5459 particles (41.8 items/g)	1.7–393.8 μm	Sheet, fibre, fragment, pellet	NA	PET, PA, PP, PE, PC, PVC, POM, PTFE, EVA, PS, PMMA, PBT, AS, PET, TPU	[36]
	11 of coastal fishermen population	3.33–13.99 μg/g	<5 mm	NA	NA	HDPE, LDPE, LLDPE, PP, PS, PET	[37]
	11 of rural farming community	6.94–16.55 μg/g	<5 mm	NA	NA	PET, PS, PP, PE, HDPE, LDPE	[38]
	26 young male students	1–36 particles/g	20–800 μm	NA	NA	PP, PET, PS, PE, PVC, PC, PA, PU	[39]
Sputum	22	18.75 − 91.75 particles/10mL	20–500 μm	NA	NA	PU, PES, Chlorinated polyethylene, alkyd varnish	[40]
Urine	6	7 particles in total	4–15 μm	Fragment, sphere	Transparent, brown, blue, green, red	PVA, PVC, PP, PE	[41]
	9	98 particles in total	0.01 nm–871 μm	Fibre, fragment	Black	PP, PA	[42]

ABS – Acrylonitrile Butadiene Styrene, CA – Cellulose Acetate, CPE – Chlorinated Polyethylene, EPS – Expanded Polystyrene, EVA – Ethylene-Vinyl Acetate, HDPE – High-Density Polyethylene, LDPE – Low Density Polyethylene, NA – not available, NC – Nitrocellulose, mm – millimetre, PA – Polyamide, PAN – Polyacrylonitrile, PBS – Phosphate-buffered Saline, PBT – Persistent Bioaccumulative Toxic, PES – Polyethersulfone, PET – Polyethylene Terephthalate, PEMA – Phenylethylmalonamide, PC – Polycarbonate, PLA – Polylactic acid, PMMA – Polymethyl Methacrylate, POM – Polyoxymethylene, PP – Polypropylene, PS – Polystyrene, PSF/PSU – Polysulfone, PU/PUR – Polyurethane, PTFE – Polytetrafluoroethylene, PVC – Polyvinyl Chloride, PVOH – Polyvinyl Alcohol, TPE – Thermoplastic Elastomers, SEBS – Styrene-Ethylene-Butylene-Styrene, μm – micrometre, μg/g – microgram per gram

**Table 3:** Summary of advantages and limitations of methodology applied in the sample matrices

Laboratory equipment and instrumentation	Type of sample	Advantages	Limitations
**Sample pre-treatment**			
10–30% KOH	Blood thrombi [18], heart [20], colectomy tissues [21], placenta [22], breastmilk [32], semen [33], stool [34,35], urine [41]	Cheap and effective that allows the isolation of microplastics from the sample. Efficiency of KOH may increase when incorporated with higher temperature at 60–70°C	Higher percentage may influence the degradation of microplastics. Time-consuming
10% KOH + CHKO_2_	Placenta [25]	CHKO_2_ increased the efficiency of the digestion process	Newly developed method is considered risky to use due to a lack of substantial supporting studies
10M KOH + sodium hypochlorite	Liver, kidney, spleen [22]	Sodium hypochlorite acts as a catalyst in increasing the efficiency of the digestion process	Sodium hypochlorite is expensive
30% H_2_O_2_	Vein [19], lung ground nodules [29], lung tissue [30], stool [39]	Readily available and relatively inexpensive. Effectively digest organic matter	Requires PPE as H_2_O_2_ is a strong oxidising agent. H_2_O_2_ may lead to formation of by-products that can interfere with the analysis of microplastics
30% H_2_O_2_ + 0.05M NaOH	Placenta [24], meconium [24]	Readily available and relatively inexpensive. Effectively digest organic matter. NaOH is cheap and can enhance the efficiency of the digestion process.	Higher percentage may influence the degradation of microplastics
30% H_2_O_2_ + 0.05M Fenton reagent	Urine [42]	Effectively digest organic matter. Fenton reagent acts as a catalyst	Expensive reagent
35% H_2_O_2_ + ZnCl_2_	Hand, hair, faces [27]	Effectively digest organic matter	ZnCl_2_ is highly toxic to the environment
HNO_3_	Placenta, infant faeces, meconium [26], stool [37,38]	Effectively digest organic matter	Highly corrosive. Can be hazardous to handle.
ZnCl_2_	Sputum [40]	Efficiency of ZnCl_2_ remains above 95% after five filtrations. Can be reused	Highly corrosive. Can be hazardous to handle. Highly toxic to the environment.
TRIS HCl buffer	Blood [17]	Works in denaturing proteins for blood sample	Newly developed method is considered risky to use due to a lack of substantial supporting studies
0.05% SDS solution +5 mM CaCl_2_ + 1 M TRIS HCl	Testis [31], semen [31]	SDS (sodium dodecyl sulfate) is a surfactant that can solubilise proteins and lipids, and it can also solubilise microplastics. The addition of CaCl_2_ can enhance the efficiency of the digestion process.	Newly developed method is considered risky to use due to a lack of substantial supporting studies.
NaOH + HNO_3_ + Protease	Lung tissues [28]	The addition of HNO_3_ and protease can enhance the efficiency of the digestion process	Newly developed method is considered risky to use due to a lack of substantial supporting studies
**Physical characterisation**			
Microscopic observation	Blood [17], thrombi [18], vein [19], colectomy [21], placenta [23,25,26], meconium [24], infant faeces [26], breastmilk [26,32], hand, hair, faces [27], lung ground nodules [29], lung tissue [30], testis [31], semen [31,33], stool [35,39], urine [41,42]	Obtain clear view of microplastic particles including their shape, size and colour. Easy to use. Non-destructive	Unable to detect the polymer type of microplastic. Prone to significant human error. Labour intensive.
Nile Red fluorescence microscopy	Liver, kidney, spleen [22]	Rapidly estimate microplastic count under the microscope. Easy to use.	Does not specify polymer composition of microplastics. Staining can conceal the original colour and surface morphology of microplastics.
SEM-EDX	Colectomy [21], lung ground nodules [29]	Able to observe any adherence of foreign particles on the microplastic sample. High resolution imaging machine that can provide detailed images of microplastics	Destructive to the sample. Time consuming and expensive.
**Chemical characterisation**			
Raman/μRaman	Thrombi [8], liver, kidney, spleen [22], placenta [23], hand, hair, faces [27], lung tissues [28], lung ground nodules [29], breastmilk [32], semen [33], stool [35,37,38], urine [41,42]	Offer precise and reliable results. Non-destructive to the microplastic particles.	Requires meticulous sample preparation. Prolonged processing time.
FTIR/μFTIR	Vein [19], colectomy tissues [21], placenta [24], meconium [24], lung ground, nodules [29], lung tissue [30], stool [34,39], sputum [40], urine [42]	Common method for analysing microplastic polymers. Offer precise and reliable results. Can detect up to 10 μm in size (for μFTIR).	Can be affected by the presence of other materials adhered on the microplastic particles. ATR-FTIR may be destructive to the surface morphology of the sample
Py-GC/MS	Blood [17], testis [31], semen [31]	Utilises various types of microplastic polymers. Offer both accuracy and high sensitivity in obtaining results. Efficient and effective approach for analysis.	Prolonged processing times. Requires high count of microplastics particles especially fibre shaped due to their low weight.
LDIR	Placenta [25,26], infant faeces [26], breastmilk [26]	Can detect up to 10 μm in size. High automation and integration	Extensive sample pre-treatment.

ATR-FTIR – Attenuated Total Reflectance-Fourier Transform Infrared Spectroscopy, CaCl_2_ – Calcium Chloride, CHKO_2_ – Potassium Formate, HNO_3_ – Nitric Acid, H_2_O_2_ – Hydrogen peroxide, KOH – Potassium Hydroxide, LD-IR – Laser Direct Infrared Spectrometry, NaOH – Sodium Hydroxide, M – molar, TRIS HCl – Tris (Hydroxymethyl) Aminomethane, mM – millimolar, PPE – Personal Protective Equipment, PY-GC/MS – Pyrolysis–Gas Chromatography Tandem Mass Spectrometry, SDS – Solution Sodium Dodecyl Sulfate Solution, SEM-EDX – Scanning Electron Microscopy/Energy Dispersive Spectroscopy, ZnCl_2_ – Zinc Chloride, μFTIR – microFourier Transform Infrared Spectrometry, μm – micrometre, μRaman – microRaman

### The Quality of studies

**Figure 3:**
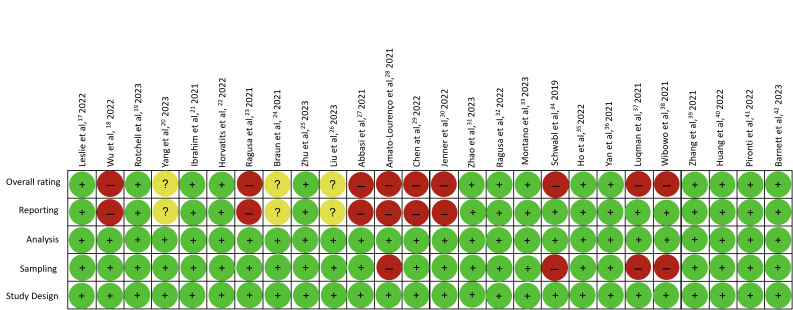
Risk of bias (RoB) adopted to this study. The RoB displays the evaluation scores for each of the four domains, as well as the overall rating for each study. A red (−) rating signifies a high risk of bias, a green (+) rating indicates a low risk of bias and a yellow (?) rating indicates an unclear risk of bias.

RoB assessment is presented in **Figure 3**. Nine studies were deemed to have high risk [18,23,27-30,34,37,38], while three were of unclear risk [20,24,26], with the remaining being low risk [17,19,21,22,25,31-33,35,36,39-42]. Studies with low risk of bias in the study design reported clear and comprehensive methodologies to identify and quantify microplastics. Four studies [28,34,37,38] have high RoB in the sampling domain due to absence of quality control measures when handling microplastics which may cause contamination from atmospheric microplastics. Additionally, six studies [18,23,27-30] have high RoB and three studies [20,24,26] with unclear risk in the reporting domain, as these studies did not report specific concentration of microplastics particles per g of tissue or ml of solution. Additionally, sample size was often mentioned as limitation in all studies.

## DISCUSSION

### Microplastics in human organ systems

Environmental plastic particles can be ingested, absorbed, digested, and removed (or remained) by the human intestines, as is similarly observed in fishes or other organisms [9,10]. The intrusion of microplastics in the digestive tract could potentially modify the gut microbiota as evidenced from various studies of human organoids [43]. Furthermore, microplastics might cause abrasions, perforations, malnutrition, mechanical injuries and even blockages of the digestive system [44]. Not only that, translocation of microplastics to other digestive organs such as the liver could occur, e.g. 11 particles from 2 cm^3^ tissue samples were reported in normal and cirrhotic liver, and interestingly more microplastics were reported in liver cirrhosis than in normal liver [22].

While adverse effects are known in marine organisms, but health effects in human are less well-studied. Hence the following discussion on health and diseases associated with microplastic is largely based on in-vitro studies, small pilot human studies and some speculation based on changes found in marine organisms. For example, notable adverse effects of microplastics to the blood vessels have included genotoxicity and cytotoxicity. In an in-vitro study, isolated human peripheral blood lymphocytes were incubated with 10–45 µm (μm) of polyethylene microplastics [45], and it was found that microplastics increased the frequency of micronucleation, nucleoplasm bridge formation and nuclear bud formation in the bloodstream. These effects have been linked to disorders such as infertility, diabetes, obesity, cardiovascular disease (coronary artery disease), chronic renal disease, cancer and neurological diseases (including Alzheimer disease and Parkinson disease) [46]. Microplastics may likely reach placenta via translocation [17], becoming vectors for transporting substances such as metals and chemicals that are endocrine disruptors [47]. They may interrupt the immune mechanisms, maternal-foetal communication, signalling between the embryo and the uterus, and trafficking of uterine dendritic cells, natural killer cells, T cells, and macrophages during a typical pregnancy [48].

Microplastic retrieved from filtered washes of hands and faces, head hairs and saliva [27] might have been attributed to the ubiquity of atmospheric microplastics but also headgears such as veils or caps, other than coming from contaminated saliva [49]. Climatic conditions may also play a role, for example, a greater quantity of microplastics was recorded in the Bushehr area of Iran due to a higher humid climate that promoted adherence of microplastics to hairs and skins [27]. Interestingly, hand skin samples have reportedly lower abundance of microplastics despite being in greater contacts with numerous sources of microplastics, and this was likely because of hand transfer and hand washing.

Microplastics have been reported in human spleen where five particles per sample of three individuals have been found [22], and despite the many vital functions of spleen, it is unclear at the moment if microplastics can cause spleen dysfunction. Alarmingly, studies have shown that microplastics could absorb and accumulate environmental contaminants, and act as vectors of bodily contaminants [49]. As microplastics can circulate in the bloodstream and potentially accumulate in various organs, including the spleen, it is possible that other potential contaminants attached to the microplastics could also be transported to the spleen. Therefore, while further research is needed to better understand the potential for microplastics to transport other pollutants to the spleen, it is possible that microplastics could play a role in the bodily accumulation of toxic chemicals.

Inhalation is the major route of translocation of environmental microplastics into the respiratory tissues [30]. Microfibre are believed to gradually accumulated with age, and the embedded microfibre in lung tissues may account for the formation of ground glass nodules; a lesion associated with chronic lung diseases. Inhaled microplastics could have negative clinical effects on the respiratory system and other organs as well [28]. For example, microfibres may accumulate in terminal bronchioles, alveolar ducts and alveoli which may eventually lead to formation of granulomas, fibrosis and chronic inflammation [50].

A recent pilot study has also reported the pollution of microplastics in human male reproductive system [31]. The exposure to microplastics may possibly cause male reproductive dysfunction as seen in mice experimented to continual contact with polystyrene which leads to a decrease in serum testosterone levels and a deterioration in sperm quality [51,52]. Even worse, a related study has unveiled nanoplastics possess more pronounced adverse effect compared to microplastics, and this includes their capability in contributing to male infertility [53].

Kidneys are particularly susceptible to water pollutants and considering that drinking water being a major source of microplastics. Advantageously, no contamination of microplastics were observed in the three samples of kidneys obtained from three healthy patients. Nonetheless, there are likely detrimental health effects of microplastics on the human kidneys, and further research is needed. Based on a study by Wang et al, exposure of human kidney proximal tubular epithelial cells (HK-2 cells) and male inbred strain mice (C57BL/6) to polystyrene microplastics resulted in mitochondrial dysfunction, endoplasmic reticulum stress, inflammation, and autophagy [54].

### Microplastics in human biological samples

Besides human organs, microplastics have been found in other human biological samples such as breastmilk, stool, sputum, or urine (**Table 2**). The presence of microplastics in these samples is due to passage storage or excretion pathways. Polypropylene, common plastic polymer utilised in various household and personal care products, was identified as the predominant form of microplastics detected in breast milk [32]. The exact mechanism by which the microplastics get into breast milk is not yet fully understood, however, it is possible that individuals, including lactating mothers, may ingest microplastics through foods or water, which are then transported to the mammary gland.

Studies have shown that microplastics is prevalent in breast milk, but, notably, the presence of microplastics in meconium and infant faeces adds another layer of concern, suggesting that exposure to these particles may continue beyond breastfeeding. Meconium, the first stool of newborns is composed of materials ingested by the foetus in the womb such as amniotic fluid and mucus. It is believed that microplastics can penetrate the foetal gut via the placenta, which is the organ that connects the foetus to the mother's womb. Since microplastics have been clearly found in the placenta [23,25] it is hypothesised that they may cross the placenta barrier and enter the foetal bloodstream, ultimately reaching the foetal gut and being excreted in the meconium [24,26]. As newborn’s digestive system matures, their stool transitions from meconium to more typical infant faeces.

For studies of microplastics in stools, dietary consumption including drinking water should be documented. Most studies recorded diet of participants for about a week before collecting their stool samples. Some studies have attempted to correlate polymers found in stools with types of diet, but despite the abundance of polymers found, there was only a moderate correlation. It is unknown if polymers found in colectomy specimens correlated with polymers found in stools. Indirect correlation seems to suggest so, with polypropylene being found in colectomy specimens in our study [24] and also stools from Schwabl et al. [34]. There are likely variations in polymer types found between geographical areas, and besides diet, other consumables may be important in explaining the difference. For example, high-density polyethylene was most common in 11 participants living in the coastal region of Surabaya, Indonesia [37] but polypropylene was commonest found in community living in rural highland village in Indonesia [38]. Besides local staple foods e.g. tempeh in Indonesia, other consumables for e.g. toothpaste and table salts have been linked to microplastics in stools.

In addition to being eliminated through stools, microplastics in the body can be excreted into the urine [41]. The sources of microplastics in urine are not entirely clear, but it is likely that they come from a variety of sources, including food packaging, personal care products and environmental contamination. Other contaminants such as Bisphenol-A (BPA) were also isolated in the human urine through gas chromatography technique coupled with mass spectrometry (GC-MS) processes [55]. The presence of BPA may explain the environmental paths of microplastics into the human urine. BPA is a chemical compound commonly used in production of certain types of plastics, including polycarbonate plastics and epoxy resins [56].

There are growing interests of finding microplastics in other bodily fluids including sputum, which is a mixture of saliva and mucus that is coughed up from the respiratory tract [40]. Interestingly, the levels of microplastics in the sputum correlated with microplastics found in dust, and dust is known to be affected by occupational background. At this moment, it is unclear the potential effects of microplastics in causing damage or inflammation within the respiratory tract.

### Physical characteristics of microplastics in human samples

#### Shape of microplastics in human samples

Microplastics can be found in different shapes depending on their sources and how they are broken down in the environment. For example, fibres or microfibres can be shed from clothing and other textiles during washing, while fragments can result from breakdown of larger plastic items, such as bottles or bags [57]. Microfibres appeared to be more durable than other types of microplastics such as fragments, films, pellets and foams. Additionally, microplastics can take on different shapes and textures as they are exposed to different environmental conditions, such as sunlight, heat, and water [36]. Findings across multiple studies indicate that microfibres could be accumulated at high levels in various human organs [21–23]. They can be as small as a few micrometres in diameter and also lightweight, which allow them to be easily inhaled and ingested by humans. Recent studies have reported excretion of two shapes, i.e. microfibres and sheets (also called, films) in stools of healthy participants and in patients with inflammatory bowel disease [36].

#### Colour of microplastics in human samples

The colour of microplastics discovered in human tissues can vary depending on a range of factors, such as polymer type and degree of degradation from human biological activities. Examples of human activities may include external actions like washing hair and hands [27] but also internal actions from digestive juices of acid and bile [21]. In general, microplastics found in human tissues tend to be transparent or translucent rather than brightly coloured. Smaller particles are more likely to be transparent or translucent [21], whereas larger particles are more opaque and coloured [27]. Furthermore, many consumer products, such as packaging and personal care items, are often made from transparent or translucent polymers [58]. Nonetheless, some studies have reported finding microplastics with various colours in human tissues, including yellow, blue, green, and red [28,32]. These colours could be attributed to additives or pigments used during the production of plastic products or from environmental factors, such as exposure to UV radiation, which can cause plastics to degrade and to discolour [5]. Overall, while the dominant colours of microplastics found in humans may vary, there is currently no evidence to suggest that the colours of microplastics have any direct effects on human health.

#### Size of microplastics in human samples

The size of microplastics is a critical factor in their ability to penetrate human tissues, with smaller particles being more likely to do so and potentially causing more harm, due to larger surface area relative to their volume, and thus increasing the potential for interactions with biological molecules. Research has shown that particles smaller than 100 μm can penetrate biological barriers and accumulate in various tissues, including the placenta [23–25]. This finding is consistent with previous observations made in blood clots, where microplastics of 2.1–26.0 μm in length were extracted [18]. Shockingly, these particles are also available in nanoscale level in urine samples, with the size of 0.01 nanometre (nm) to 0.60 μm [42], raising additional concerns about the potential health effects that these minuscule particles may bring along the human organ systems before being eliminated through biological processes. At this tiny size, their behaviour tends to exhibit complex interactions with cellular membranes of which their transport mechanisms are still being studied [59]. Several factors, such as the tissue type and location of exposure, also determine the precise size of microplastics that can penetrate human tissue. For instance, microplastics were seen more abundantly in soft tissues than hard tissues [21,22,28]. Soft tissues are composed of cells and extracellular matrix, and include connective tissue, muscle tissue, nervous tissue, and epithelial tissue. Notably, larger microplastics exceeding 4000 μm have been observed in human stool samples [36].

#### Polymer of microplastics in human samples

Microplastics have been identified as a potential vector for pollutants and chemicals, facilitating their entry into human tissues. Chemicals could leach either from plastics themselves or from chemicals absorbed from the environment. Studies have shown that microplastics contained a range of toxic chemicals, such as phthalates and BPA [55,60]. These have been linked to various health problems, including cancer, developmental disorders, and reproductive problems [55]. Other studies have shown that polypropylene and polyethylene microplastics can accumulate in various human tissues but are more abundant in digestive tract, placenta and lungs [21,24,29]. The reason for greater abundance of the polymers polyethylene in these human organs compared to others is unknown but these polymers are commonly found in consumer goods, such as food packaging, cosmetics, and textiles [57].

### Potential pathway of microplastics into human

Microplastics can enter the body through inhalation and ingestion. Contaminated food, water, and polluted air are common sources of microplastics [61]. Studies have shown that inhalable microplastics particles with a size of less than 10 μm [28] can enter the respiratory system and intrathoracic cavity of humans, with an estimated annual inhalation exposure of 53 700 particles per person [61], assuming an inhalation rate of 15 m^3^/d [62]. Even though the mucociliary function in the respiratory system can be effective barrier against intruding particles such as microplastics, a small number of microplastics can still persist in the lungs and cause certain bodily reactions [52]. For instance, inflammation could happen due to suspension of pollutants such as polycyclic aromatic hydrocarbons and metals on the hydrophobic surfaces of atmospheric microplastics [63]. Microplastics are ubiquitous in the atmosphere due to their small size and various meteorological factors [64], and their abundance varies across different countries, with megacities in China [65] reporting a higher abundance of microplastics than urban and suburban areas in Indonesia [66]. Exact reasons behind the differences between the two countries are unclear but lack of a standardised sampling method may be a reason.

Being rich in vital nutrients, seafood is essential for human nutrition and global food security [67]. However, microplastics are abundant in seafood. In commercial fish (*Atule mate*, *Crenimugil seheli*, *Sardinella fimbriata*, and *Rastrelliger brachysoma*) from Malaysia Northwest Peninsular seawater, microplastics were present in 100% of the samples with *S. fimbriata* has the highest average microplastic abundance at 6.5 ± 4.3 microplastics per organism [9]. Microplastics are also abundant in marine dried fish products that are widely consumed in Asian countries including Taiwan, Japan, Thailand, South Korea and Sri Lanka [68,69].

Microplastics can be present in abundance in drinking water, especially bottled ones. The concentration of microplastics in bottled water varies depending on the country and the brand, with some brands containing high levels of microplastics (>25 μm) [70]. The morphology of microplastics found in bottled water may include fragments and fibres of different lengths [71,72] and the predominant polymers found in bottled water were polyethylene, polystyrene and polyethylene terephthalate [73]. An average adult, with a body weight of 61.57 kg, may consume around 0.09–0.19 million microplastics particles per day from bottled water [74]. This estimation is based on the average daily intake (EDI) of microplastics in bottled water. The amount of microplastics consumed may vary based on the type and quality of bottled water and the individual's drinking habits. Therefore, it may be essential to monitor the levels of microplastics in bottled water and to take measures to reduce their presence to safeguard the public health.

### Potential health effects of microplastics

At the cellular and molecular level, microplastics can induce oxidative stress in skeletal muscle by generating reactive oxygen (ROS) as seen when PS subjected to satellite cell [75]. Microplastics of the size 0.5 μm could be phagocytosed by macrophages, leading to the increase levels of ROS [76], disrupting mitochondrial kinetic homeostasis [77]. Additionally, with the increasing ROS, it may also lead to lipid peroxidation, damaging other lipid-containing structures such as cell membranes [78]. In another study, a cytotoxic effect was observed after introducing PVC microplastics into a simulated digestive tract model. The gene expression levels of DDIT3 and OXR1 significantly increased, indicating that the Caco-2 cell membrane was under oxidative stress, contributing to the observed cytotoxicity [79].

### Limitations and advantages of each methodology employed

Limitations and advantages of existing methodologies to detect microplastics is presented in **Table 3**. Chemicals used for sample digestion in most studies include 10–30% potassium hydroxide (KOH), 30% hydrogen peroxide (H_2_O_2_), nitric acid (HNO_3_), and zinc chloride (ZnCI_2_). Catalysts such as potassium formate (CHKO_2_), Fenton reagent and sodium hydroxide (NaOH) were also employed to improve efficiency in addition of chemical digestion of HNO_3_, ZnCI_2_ and H_2_O_2_. Commonly observed techniques for processing diverse human samples such as blood, tissues, stools, semen and urine, involve the utilisation of a 10% KOH for digestion. This readily available solution is applied to facilitate the breakdown and preparation of these samples for further analysis or testing [80]. Their efficiency may also increase when incorporating with higher temperatures at 60–70°C [81]. Additionally, KOH is a common choice for digesting various other types of samples and matrices including fish [9], shellfish [10] and even sediments [82]. Meanwhile, a less documented method involving the use of TRIS HCl buffer along with a combination of sodium dodecyl sulphate solution and calcium chloride (CaCl_2_), as well as additional HNO_3_ and protease as catalysts has been shown to yield microplastics [17,28,31]. However, this method lacks significant supporting studies, as it is not widely employed yet.

Physical characterisation involves the optical microscopy observation which is widely applied in across studies as it is easy to use and to classify microplastics particles according to their colour, shape and size. Following this, two reported studies [21,29] has further investigated their samples with Scanning Electron Microscopy/Energy Dispersive Spectroscopy (SEM-EDX) to visualise microplastics at high magnifications [83]. Furthermore, there are multiple methods available for detecting polymer composition of microplastics including Raman/(μRaman) spectroscopy, fourier transform infrared spectroscopy (FTIR)/micro-FTIR (μFTIR), pyrolysis-gas chromatography mass spectrometry (Py-GC/MS), and laser-induced laser direct infrared spectroscopy (LDIR). Each of this approach has its own advantages and limitations mentioned in **Table 3**.

### Potential sources of contamination

Despite the variety of approaches for detecting microplastics in human sample, there could be potential sources of contamination in each methodology if not handled thoroughly. Several studies do not report on the preparation and use of blanks in their laboratory processes [20,24,26]. Blanks are being performed by mimicking the same process of laboratory experiment to identify potential contaminants [84]. For instance, a blank chemical solution without any matrix should be processed alongside the digestion of samples [21]. During physical characterisation using stereomicroscope, another set of blank either dry or wet filter paper should be placed near the working environment until the step is over [85,86]. These blanks are then observed for atmospheric microplastics that may contaminate the samples during processing. Conducting these steps in future studies would generate more reliable and accurate results as the presence of microplastics in the atmosphere is undeniable and could contaminate the samples.

## CONCLUSIONS

Microplastics have been detected in more than half of human organ systems. These microplastics may be classified according to their morphology, colours, size, and type of polymers. The exposure routes to microplastics are likely from inhalation and ingestion with subsequent translocation into the organ systems. We have also systematically evaluated the risk of bias associated with each research methodology employed to extract microplastics in human samples, providing a comprehensive list of the strengths and limitations inherent to each approach. However, correlation between microplastics with their sources remains poorly studied, and likewise correlations with adverse health effects. More research is needed to better understand the long-term effects of microplastics on human health and ways to mitigate exposure to microplastics. Future studies should employ strict control contamination procedures during handling and processing of the samples to avoid atmospheric microplastics.

## Additional material


Online Supplementary Document
