# Supplementary material for: Detection of microplastics in human tissues and organs: A scoping review
Source: J Glob Health. 2024 Aug 23;14:04179. doi: 10.7189/jogh.14.04179 (PMC11342020; doi:10.7189/jogh.14.04179)
Supplement: Online Supplementary Document [file jogh-14-04179-s001.pdf]

Online Supplementary Material

Table S1. Preferred Reporting Items for Systematic reviews and Meta-Analyses extension for Scoping Reviews (PRISMA-ScR) Checklist

SECTION	ITEM	PRISMA-ScR CHECKLIST ITEM	REPORTED ON PAGE #
**TITLE**			
Title	1	Identify the report as a scoping review.	1
**ABSTRACT**			
Structuredsummary	2	Provide a structured summary that includes (as applicable): background, objectives, eligibility criteria, sources of evidence, charting methods, results, and conclusions that relate to the review questions and objectives.	1
**INTRODUCTION**			
Rationale	3	Describe the rationale for the review in the context ofwhat is already known. Explain why the review questions/objectives lend themselves to a scoping review approach.	2
Objectives	4	Provide an explicit statement of the questions and objectives being addressed with reference to their key elements (e.g., population or participants, concepts, andcontext) or other relevant key elements used to conceptualize the review questions and/or objectives.	2
**METHODS**			
Protocol and registration	5	Indicate whether a review protocol exists; state if and where it can be accessed (e.g., a Web address); and ifavailable, provide registration information, including theregistration number.	3
Eligibility criteria	6	Specify characteristics of the sources of evidence usedas eligibility criteria (e.g., years considered, language, and publication status), and provide a rationale.	3
Informationsources*	7	Describe all information sources in the search (e.g., databases with dates of coverage and contact with authors to identify additional sources), as well as thedate the most recent search was executed.	3
Search	8	Present the full electronic search strategy for at least 1 database, including any limits used, such that it could be repeated.	3
Selection of sources of evidence†	9	State the process for selecting sources of evidence (i.e., screening and eligibility) included in the scoping review.	3
Data charting process‡	10	Describe the methods of charting data from the included sources of evidence (e.g., calibrated forms or forms thathave been tested by the team before their use, and whether data charting was done independently or in duplicate) and any processes for obtaining and confirming data from investigators.	3

Data items	11	List and define all variables for which data were soughtand any assumptions and simplifications made.	3
Critical appraisal of individual sources of evidence§	12	If done, provide a rationale for conducting a critical appraisal of included sources of evidence; describe the methods used and how this information was used in anydata synthesis (if appropriate).	3-4
Synthesis of results	13	Describe the methods of handling and summarizing the data that were charted.	4
**RESULTS**			
Selection ofsources of evidence	14	Give numbers of sources of evidence screened, assessed for eligibility, and included in the review, with reasons for exclusions at each stage, ideally using a flow diagram.	4
Characteristics of sources of evidence	15	For each source of evidence, present characteristics for which data were charted and provide the citations.	4
Critical appraisal within sources of evidence	16	If done, present data on critical appraisal of includedsources of evidence (see item 12).	4
Results of individual sourcesof evidence	17	For each included source of evidence, present the relevant data that were charted that relate to the review questions and objectives.	4 - 11
Synthesis of results	18	Summarize and/or present the charting results as theyrelate to the review questions and objectives.	4
**DISCUSSION**			
Summary ofevidence	19	Summarize the main results (including an overview of concepts, themes, and types of evidence available), link to the review questions and objectives, and consider the relevance to key groups.	5-12
Limitations	20	Discuss the limitations of the scoping review process.	14
Conclusions	21	Provide a general interpretation of the results with respect to the review questions and objectives, as well as potential implications and/or next steps.	14
**FUNDING**			
Funding	22	Describe sources of funding for the included sources of evidence, as well as sources of funding for the scoping review. Describe the role of the funders of the scoping review.	14-15

JBI = Joanna Briggs Institute; PRISMA-ScR = Preferred Reporting Items for Systematic reviews and Meta-Analysesextension for Scoping Reviews.

* Where *sources of evidence* (see second footnote) are compiled from, such as bibliographic databases, social mediaplatforms, and Web sites.

† A more inclusive/heterogeneous term used to account for the different types of evidence or data sources (e.g.,quantitative and/or qualitative research, expert opinion, and policy documents) that may be eligible in a scoping review as opposed to only studies. This is not to be confused with *information sources* (see first footnote).

‡ The frameworks by Arksey and O'Malley (6) and Levac and colleagues (7) and the JBI guidance (4, 5) refer to theprocess of data extraction in a scoping review as data charting.

§ The process of systematically examining research evidence to assess its validity, results, and relevance before using it to inform a decision. This term is used for items 12 and 19 instead of "risk of bias" (which is more applicable to systematic reviews of interventions) to include and acknowledge the various sources of evidence that may be usedin a scoping review (e.g., quantitative and/or qualitative research, expert opinion, and policy document).

From: Tricco AC, Lillie E, Zarin W, O'Brien KK, Colquhoun H, Levac D, et al. PRISMA Extension for Scoping Reviews (PRISMAScR): Checklist and Explanation. Ann Intern Med. 2018;169:467–473. doi: 10.7326/M18-0850.

Appendix S1. The search strategy

PubMed

#1 (("microplastics"[MeSH Terms] OR "microplastics"[All Fields] OR "microplastic"[All Fields]) AND "human"[Title/Abstract]) OR (("microplastics"[MeSH Terms] OR "microplastics"[All Fields] OR "microplastic"[All Fields]) AND "human organ"[Title/Abstract]) OR (("microplastics"[MeSH Terms] OR "microplastics"[All Fields] OR "microplastic"[All Fields]) AND "human tissue"[Title/Abstract]) OR (("microplastics"[MeSH Terms] OR "microplastics"[All Fields] OR "microplastic"[All Fields]) AND "human health effects"[Title/Abstract]) OR ((("environment"[MeSH Terms] OR "environment"[All Fields] OR "environmental"[All Fields] OR "environmentally"[All Fields] OR "environmentals"[All Fields]) AND ("microplastics"[MeSH Terms] OR "microplastics"[All Fields] OR "microplastic"[All Fields]) AND ("pathway"[All Fields] OR "pathway s"[All Fields] OR "pathways"[All Fields])) AND "human"[Title/Abstract]) OR (("microplastics"[MeSH Terms] OR "microplastics"[All Fields] OR "microplastic"[All Fields]) AND "human cancer"[Title/Abstract])

#2 (("microplastics"[MeSH Terms] OR "microplastics"[All Fields] OR "microplastic"[All Fields]) AND "human biological sample"[Title/Abstract]) OR (("microplastics"[MeSH Terms] OR "microplastics"[All Fields] OR "microplastic"[All Fields]) AND "human"[Text Word]) OR (("microplastics"[MeSH Terms] OR "microplastics"[All Fields] OR "microplastic"[All Fields]) AND "in vivo"[Title/Abstract])

#3 #1 OR #2

Web of Science

#1 ((((TI=(microplastic in human)) OR TI=(microplastic in human body)) OR TI=(microplastic human organ)) OR TI=(environmental microplastic human)) OR ALL=(microplastic human)

#2 ((((TI=(microplastic in human)) OR TI=(microplastic in human body)) OR TI=(microplastic human organ)) OR TI=(environmental microplastic human)) OR ALL=(microplastic human)

#3 #1 OR #2

Table S2. Risk of Bias (RoB) Assessment Tool Adopted to This Study

Domain	No	Question	Answer	Notes	Rating (high, low, unclear)
Internal validity					
Appropriateness of study design to the research objective	1	Is the design appropriate for the questions of the study?			
Sampling					
Sample method	2	Has the method been used in other studies?			
	3	Is the method validated?			
	4	Are there precautions in place to protect further contamination of the sample?			
Sample location	5	Is there a rationale available?			
	6	Is the location appropriate?			
Sample randomization	7	Is the sampling method guarantying randomization of the sample?			
Use of procedural blank samples	8	Are the results of the procedural blank samples reported?			
Use of replicate samples	9	Is the study using replicate samples?			
	10	How many?			
Analysis					
Particles extraction method	11	Is the method used by other studies?			
	12	Is the method validated?			
Particles identification method	13	Is the method one of the four validated methods?			
Amount of sample analysed for composition.	14	How much of the sample has been analysed?			
Particle composition match to the library of choice	15	Is the match > or < 60% match?			

Library of choice (type, kind)	16	Is the library made by the lab or is it a commercial library?			
	17	Is one library or more being used?			
Statistical analysis	18	Is the statistical analysis appropriate for the sample?			
Interpretation	19	Has the interpretation of the results been based on the outcomes of the analysis?			
Quality of reporting					
Methodology	20	Have the methods used in the study been reported in detail?			
Limitations	21	Have the study recognized limitations?			
				Overall score	
